# Standard exercise stress testing attenuates peripheral microvascular function in patients with suspected coronary microvascular dysfunction

**DOI:** 10.1186/s13102-021-00246-8

**Published:** 2021-02-27

**Authors:** Massimo Nardone, Steven Miner, Mary McCarthy, Heather Edgell

**Affiliations:** 1 School of Kinesiology and Health Science, York University, 355 Norman Bethune College, Toronto, Ontario Canada; 2 Southlake Regional Health Center, Newmarket, Ontario Canada

**Keywords:** Peripheral arterial tonometry, EndoPAT, Acute exercise, Augmentation index, Microvascular, Reactive hyperemia

## Abstract

**Background:**

The effect of exercise on the microvasculature of patients with suspected coronary microvascular dysfunction (CMD), assessed by reactive hyperemia peripheral arterial tonometry (RH-PAT; EndoPAT), is unknown. The present study aimed to determine if standard clinical exercise stress testing (GXT) affected peripheral microvascular function, as determined by the reactive hyperemia index (RHI and LnRHI), in patients with suspected CMD.

**Methods:**

In a cross-sectional study, patients (*n* = 76) were grouped based on whether the GXT was performed; 1) prior to (exercisers; *n* = 30), or 2) after the vascular assessment (non-exercisers; *n* = 46). Patients with an adenosine index of microvascular resistance > 25, adenosine coronary flow reserve (CFR) < 2.0, and/or acetylcholine CFR < 1.5 were considered to have CMD (*n* = 42). RHI and LnRHI quantified finger pulse amplitude hyperemia following 5 min of forearm ischemia.

**Results:**

LnRHI was lower in patients with CMD compared to patients without CMD, while LnRHI was also lower in exercisers compared to non-exercisers (LnRHI: CMD Non-Exercisers: 0.63 ± 0.25; CMD Exercisers: 0.54 ± 0.19; No CMD Non-Exercisers: 0.85 ± 0.23; No CMD Exercisers: 0.63 ± 0.26; Condition and Exercise Main Effects: Both *P* < 0.01). In patients who did not exercise prior to the vascular assessment, the receiver operating characteristic curve (ROC) for LnRHI to predict CMD was 0.76 (95% CI: 0.62–0.91; *P* < 0.01). However, in patients who performed exercise prior to the vascular assessment, the ROC for LnRHI to predict CMD was 0.60 (95% CI: 0.40–0.81; *P* = 0.34).

**Conclusions:**

CMD is associated with impaired peripheral microvascular function and preceding acute exercise is associated with further reductions of LnRHI. Further, acute exercise abolished the capacity for RH-PAT to predict the presence of CMD in patients with chest pain and non-obstructive coronary arteries. RH-PAT measurements in patients with suspected CMD should not be conducted after exercise has been performed.

## Background

Reactive hyperemia peripheral arterial tonometry (RH-PAT) is emerging as a novel measure of microvascular endothelial function, and is obtained by quantifying the hyperemic response within the finger microvasculature following brief ischemia [1, 2]. Peripheral microvascular function, using RH-PAT, predicts major adverse cardiovascular events in both health and disease [3–5]. In addition, peripheral endothelial dysfunction has been shown to relate to the function of coronary vasculature [6–8], implying that measuring peripheral endothelial function may be a time- and cost-efficient analysis to estimate coronary function in patients with suspected cardiovascular disease. Such analyses may be particularly relevant in patients with coronary microvascular dysfunction (CMD), a diseased characterized by abnormal coronary microvascular vasomotion and increased rates adverse cardiac outcomes [9–11]. In support of this, we have recently demonstrated that poor peripheral microvascular function, using RH-PAT, identifies patients with CMD [6], supporting its utility within clinical settings.

Despite the growing interest in RH-PAT assessments, factors known to influence RH-PAT remain poorly defined relative to other established measures of endothelial function, such as flow-mediated dilation [12, 13]. In particular, the impact of acute exercise on RH-PAT in patients with CMD is currently unknown. Studies quantifying endothelial function using flow-mediated dilation in healthy participants have highlighted that acute exercise briefly attenuates endothelial function, the magnitude of which is dependent on the duration, intensity, and type of exercise, as reviewed by Dawson et al. [14]. Unfortunately, these observations cannot necessarily be applied to RH-PAT, given that both flow-mediated dilation and RH-PAT are poorly correlated [15] and can be differentially influenced [15–17].

In addition, it is currently unclear whether patients with CMD experience an exaggerated reduction of endothelial function following exercise. Lower resting endothelial function is thought to lead to greater impairments of post-exercise vascular function [14], relevant considering that patients with CMD often present with reduced endothelial function [6, 18, 19]. Lastly, whether post-exercise alterations in endothelial function translate into diminished predictive capacity of RH-PAT for identifying patients with CMD remains unknown. Therefore, the present study aimed to: 1) confirm that patients with clinically diagnosed CMD exhibit impaired peripheral microvascular function, 2) determine if standard clinical exercise stress testing, commonly conducted within clinical practice, acutely affects peripheral microvascular function in patients with suspected CMD, and 3) determine if exercise abolishes the predictive capacity of RH-PAT. We hypothesized that patients would have reduced peripheral microvascular function which would be further impaired by exercise such that RH-PAT measurements could no longer be used to predict the presence of CMD.

## Methods

### Patient selection

Seventy-six patients with suspected coronary microvascular disease (CMD) who attended the Cardiovascular Integrative Physiology Clinic at Southlake Regional Health Centre were recruited for this study. All non-exercisers included in this study also appear in Nardone et al. 2020 [6]. Subjects were included in this study if they underwent reactive hyperemia peripheral arterial tonometry (RH-PAT) assessments and coronary reactivity testing, to functionally assess peripheral and coronary microvascular function, respectively. Coronary reactivity testing was conducted if participants were experiencing typical or atypical chest pain and had angiographic evidence of normal coronary arteries (stenosis < 50%). Detailed exclusion criteria for the current study have been described previously [6]. Briefly, these include: 1) hypertrophic cardiomyopathy, 2) coronary epicardial stenosis, 3) recent coronary artery intervention or bypass grafting, 4) heart failure (EF < 40%), and 5) pulmonary hypertension induced by exercise. All procedures were approved by the Research Ethics Boards of Southlake Regional Health Centre and York University.

### Experimental protocol

In a cross-sectional design, patients were grouped based on whether an exercise stress test was performed 1) prior to, or 2) following the vascular assessment. The exercise assessment consisted of a symptoms-limited maximal treadmill exercise stress test (GXT) using the Bruce protocol with concurrent 12-lead electrocardiography. The Duke Treadmill Score (DTS) was subsequently calculated, using the formula: DTS = Exercise time – (5 x Max exercise ST) – (4 x Angina Index), as previously ascribed for risk classification [20]. Maximum exercise ST depression was the maximal depression present during exercise, where a maximal ST segment depression of ≥0.1 mV was considered relevant myocardial ischemia. The angina index was ranked 0–2; 0 defined as no chest pain, 1 defined as non-limiting chest pain, and 2 defined as limiting chest pain. Heart rate and blood pressure were recorded both at rest and during maximal exercise. The time duration between the GXT and the vascular assessment was documented.

Prior to the vascular assessment, blood pressure was manually obtained, and height and body mass were recorded. Non-invasive assessment of peripheral microvascular function was conducted using reactive hyperemia peripheral arterial tonometry (RH-PAT) (EndoPat, Itamar Medical, Israel). Briefly, an applanation tonometry cuff was placed on the index finger of both hands. A blood pressure cuff was positioned on the right arm, immediately distal to the elbow joint. Five minutes of baseline recordings preceded 5 min of forearm ischemia, which involved supra-systolic cuff inflation to ≥200 mmHg. Subsequently, the blood pressure cuff was quickly deflated to allow reperfusion of the distal limb for an additional 5 min. The hyperemic response was quantified as the reactive hyperemic index (RHI) and the natural logarithm of RHI (LnRHI) (due to non-normal distribution of RHI) using automated algorithms of the EndoPat device [21]. More than 100 pulse amplitude waveforms of entire cardiac cycles were used during the baseline phase to determine augmentation index (AI), a measure of arterial stiffness. Further, due to the temporal relationship between heart rate and augmentation index [22], AI was also calculated at a controlled heart rate of 75 beats per minute (AI@75 bpm).

Approximately 5 months later, patients underwent coronary reactivity testing using the Doppler guidewire method, as previously described [6, 23]. In brief, a 0.014-in pressure-temperature sensor-tipped Doppler guidewire was advanced into the left anterior descending coronary artery (LAD), which simultaneously measured proximal (Pa) and distal (Pd) coronary pressures, and the mean transit time of coronary flow (Tmn) via thermodilution [24]. The fractional flow reserve (FFR) was collected to confirm patients did not have significant coronary epicardial stenosis. An FFR < 0.80 was considered hemodynamically significant coronary epicardial stenosis, and if present, patients were excluded. The Pa, Pd, and Tmn were collected at rest and during intravenous adenosine (140μg/kg/min) and intracoronary acetylcholine (20 μg bolus + 100 μg slow injection over 90 s). During pharmacological hyperemia, the coronary flow reserve (CFR), and the index of microvascular resistance (IMR) were calculated as CFR = Tmn_baseline_ / Tmn_hyperemia_, and IMR = Pd_hyperemia_ x Tmn_hyperemia_ [25]. As previously described by our group [6], an adenosine IMR > 25, adenosine CFR < 2.0, and/or acetylcholine CFR < 1.5 was used as the diagnostic criteria for CMD.

### Data and statistical analysis

Anthropometric data, GXT data, coronary reactivity testing data, and RH-PAT data were compared using a two by two factorial ANOVA. Levene’s test of equality of variances was conducted prior to all ANOVA analyses. If a significant interaction was observed from the ANOVA, post hoc testing was conducted with the Bonferroni adjustment. The time between the GXT and the vascular assessment was compared between groups using an independent samples t-test. Medication regimen was grouped based on if subjects were taking; 1) any anti-hypertensive medications (i.e. one or more of the following: beta-adrenergic antagonist, angiotensin converting enzyme (ACE) inhibitor, angiotensin receptor blocker (ARB), diuretics, or a calcium channel blocker), and 2) any anti-cholesterol medications (i.e. one or more of the following: statin, or cholesterol absorption inhibitors). Medication usages were compared between groups using Chi squared testing. Further, use of either medication (i.e. anti-hypertensive and anti-cholesterol medications) and fasted state were utilized as covariates for ANCOVA analysis. To investigate the effect of time between the GXT and the vascular assessment, Pearson correlations between the LnRHI and the time between tests in exercisers with or without CMD were conducted. Lastly, to determine if exercise effects the capacity for RH-PAT to predict the presence of CMD, receiver operating characteristic (ROC) curve analysis was conducted to determine the ROC area under the curve (ROC_AUC_) in both exercisers and non-exercisers. All statistical analyses were performed using IBM SPSS Statistics 23 (Armonk, NY). Parametric data is presented as Mean ± SD, while non-parametric data is presented as Count (%). Significance was defined as *P* < 0.05.

## Results

**Fig. 1:**
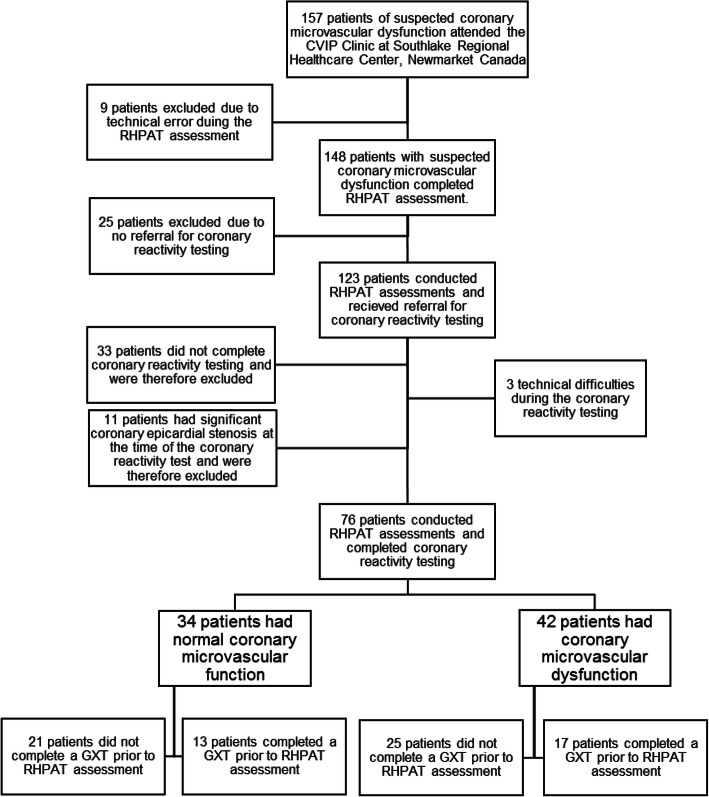
Schematic of patients recruited from the Cardiovascular Integrative Physiology Clinic (CVIP) at Southlake Regional Health Centre. RH-PAT: Reactive Hyperemia Peripheral Arterial Tonometry; GXT Graded Exercise Test

Between May 2017 and April 2019, 157 patients with suspected coronary microvascular dysfunction (CMD) attended the Cardiovascular Integrated Physiology Clinic at Southlake Regional Healthcare Centre for reactive hyperemia peripheral arterial tonometry (RH-PAT) assessments. After exclusion of patients who experienced technical errors during RH-PAT assessments (*n* = 9), patients who did not receive a referral for coronary reactivity testing (*n* = 25), patients who did not complete coronary reactivity testing (*n* = 33), patients who had hemodynamically significant coronary epicardial stenosis (*n* = 11), and patients who had technical difficulties during the coronary reactivity testing (*n* = 3), the sample consisted of 34 patients with normal coronary microvascular function and 42 patients with CMD (Fig. 1).

In patients with CMD, 25 patients did not exercise prior to assessment, and 17 patients exercised prior to assessment. In those without CMD, 21 patients did not exercise prior to assessment, and 13 patients exercised prior to assessment. There were no differences in anthropometrics, resting blood pressure, anti-cholesterol medications use, or fasted state between all groups (*P* > 0.05; Table 1). Resting heart rate was elevated in exercisers compared to non-exercisers (*P* = 0.02; Table 1). Prior to coronary reactivity testing, a greater proportion of patients with confirmed CMD were taking anti-hypertensive medications compared to patients without CMD (*P* < 0.05; Table 1). Statistical analyses for the adenosine IMR, adenosine CFR, and acetylcholine CFR were not conducted due to these variables being utilized as diagnostic criteria. However, the acetylcholine IMR was greater in patients with CMD compared to patients without CMD (P < 0.05; Table 2). Additionally, there was no difference in coronary microvascular function between non-exercisers and exercisers in both conditions (*P* > 0.05; Table 2).

**Table 1:** Anthropometrics, hemodynamics, medication use, and fasted state in patients with or without coronary microvascular dysfunction (CMD)

	No CMD			CMD		
	All Patients	Non-EX	Exercisers	All Patients	Non-EX	Exercisers
n	34	21	13	42	25	17
Sex, male, n (%)	13 (38)	7 (33)	6 (46)	13 (31)	8 (32)	5 (29)
Age (years)	55 ± 13	58 ± 13	51 ± 13	60 ± 13	62 ± 13	57 ± 11
Body mass (kg)	81.8 ± 19.0	76.8 ± 18.4	90.0 ± 17.6	78.1 ± 15.4	79.1 ± 14.6	76.7 ± 16.8
Height (m)	1.69 ± 0.10	1.67 ± 0.10	1.73 ± 0.08	1.66 ± 0.10	1.66 ± 0.08	1.67 ± 0.12
BMI (kg/m^2^)	29 ± 6	27 ± 6	30 ± 6	28 ± 4	29 ± 4	27 ± 5
Resting SBP (mmHg)	124 ± 15	124 ± 18	124 ± 10	123 ± 12	126 ± 12	117 ± 11
Resting DBP (mmHg)	75 ± 8	76 ± 8	74 ± 8	76 ± 9	77 ± 9	74 ± 9
Resting HR (bpm)	67 ± 12	62 ± 9	74 ± 13^a^	68 ± 13	65 ± 13	72 ± 12^a^
Medication use, n (%)						
Anti-Hypertensive	21 (62)	13 (62)	8 (62)	35 (83)†	21 (84)	14 (82)
Anti-Cholesterol	19 (56)	12 (57)	7 (54)	26 (62)	16 (64)	10 (59)
Fasted, n (%)	4 (12)	3 (14)	1 (8)	11 (26)	8 (32)	3 (18)

*Non-EX* Non-Exercisers, *CMD* Coronary Microvascular Dysfunction, *SBP* Systolic Blood Pressure, *DBP* Diastolic Blood Pressure, *HR* Heart Rate, *BMI* Body Mass Index. ^a^ indicates a significant exercise effect. † indicates a significant effect of CMD. Mean ± SD

**Table 2:** Coronary flow and resistance responses to coronary infusions of adenosine and acetylcholine in patients with or without coronary microvascular dysfunction (CMD) undergoing coronary reactivity testing

	No CMD			CMD		
	All Patients	Non-EX	Exercisers	All Patients	Non-EX	Exercisers
Adenosine						
IMR	16.3 ± 4.4	15.8 ± 4.6	17.3 ± 4.0	26.2 ± 13.3	27.3 ± 15.8	24.6 ± 8.5
CFR	4.0 ± 1.6	4.1 ± 1.9	3.7 ± 1.1	2.8 ± 1.6	2.7 ± 1.9	2.8 ± 1.0
Acetylcholine						
IMR	24.0 ± 11.6	24.4 ± 11.9	23.2 ± 11.3	39.7 ± 21.7^a^	40.1 ± 19.6	39.2 ± 25.4
CFR	2.9 ± 1.4	2.7 ± 1.6	3.1 ± 1.0	1.9 ± 1.3	1.6 ± 0.8	2.3 ± 1.7

*Non-EX* Non-Exercisers, *CMD* Coronary Microvascular Dysfunction, *CFR* Coronary Flow Reserve, *IMR* Index of Microvascular Resistance. ^a^ indicates a significant effect of CMD. Mean ± SD

Five patients in both non-exerciser groups did not complete the graded exercise stress test at any time as a part of their normal clinical care (GXT; Table 3). In patients who were able to exercise, there were no differences in symptoms of exertional chest pain, exercise-induced myocardial ischemia, the Duke Treadmill Score between groups, or hemodynamics at peak exercise (*P* > 0.05; Table 3). Although there was an interaction between groups and exercise duration (Interaction: *P* = 0.048; Table 3), post hoc testing indicated that there were no significant differences in exercise duration between groups (P > 0.05). In exercisers, the time between the GXT and the vascular assessment was not different between patients with and without CMD (*P* > 0.05; Table 3).

**Table 3:** Symptoms-limited maximal exercise testing data in patients with or without coronary microvascular dysfunction (CMD)

	No CMD		CMD	
	Non-EX	Exercisers	Non-EX	Exercisers
n	18	13	20	17
LBBB, n (%)	1 (6%)	0 (0)	2 (10)	0 (0)
Time between tests (mins)		68 ± 38		68 ± 29
Exercise duration (mins)	8.36 ± 3.11	7.36 ± 1.56	7.26 ± 3.24	7.32 ± 3.32
Chest pain symptoms, n (%)				
Non-Limiting	2 (11)	3 (23)	5 (25)	5 (29)
Limiting	1 (6)	3 (23)	5 (25)	3 (18)
ST Depression, n (%)	10 (56)	8 (62)	11 (55)	5 (29)
Duke Treadmill Score (au)	3 ± 5	1 ± 5	0 ± 7	3 ± 5
Hemodynamics				
Peak HR (bpm)	140 ± 21	137 ± 23	137 ± 21	136 ± 18
Peak SBP (mmHg)	151 ± 18	154 ± 18	151 ± 12	138 ± 20
Peak DBP (mmHg)	78 ± 11	76 ± 9	80 ± 10	76 ± 9

*Non-EX* Non-Exercisers, *CMD* Coronary Microvascular Dysfunction, *DTS* Duke Treadmill Score, *LBBB* left bundle branch block, *HR* heart rate, *SBP* systolic blood pressure, *DBP* diastolic blood pressure. Time between tests represents the time between the exercise stress test and peripheral arterial tonometry in exercisers. No significant effect of exercise or CMD for all variables (All *P* > 0.05). Mean ± SD. Count (%)

**Fig. 2:**
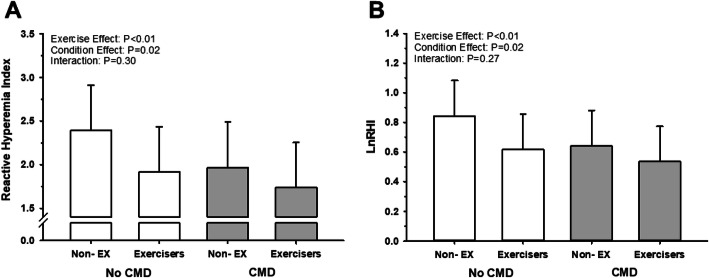
Adjusted reactive hyperemia index (RHI; **a**) and natural logarithm of the RHI (LnRHI; **b**) in patients with or without coronary microvascular dysfunction (CMD). Patients were separated into groups who did and did not exercise prior to vascular assessment. White bars represent patients without CMD. Grey bars represent patients with CMD. Non-EX indicates patients who did not exercise prior to assessment. Mean ± SD

**Fig. 3:**
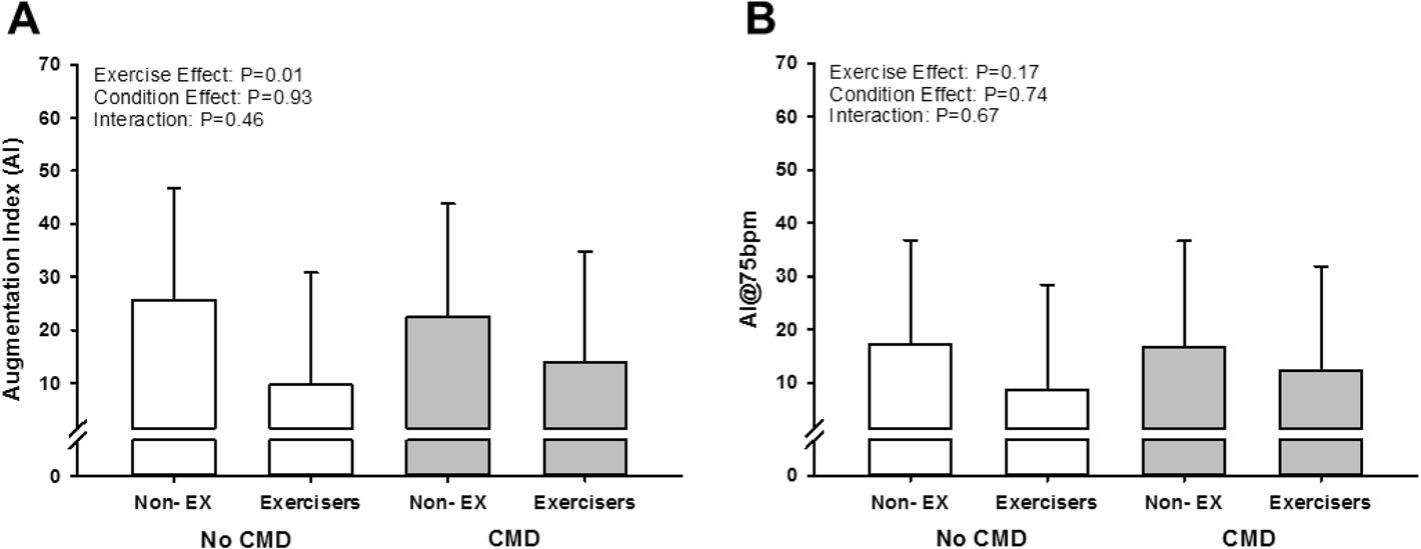
Adjusted augmentation index (AI; **a**) and augmentation index at 75 bpm (AI@75 bpm; **b**) in patients with or without coronary microvascular dysfunction (CMD). Patients were separated into groups who did and did not exercise prior to vascular assessment. White bars represent patients without CMD. Grey bars represent patients with CMD. Non-EX indicates patients who did not exercise prior to assessment. Mean ± SD

**Fig. 4:**
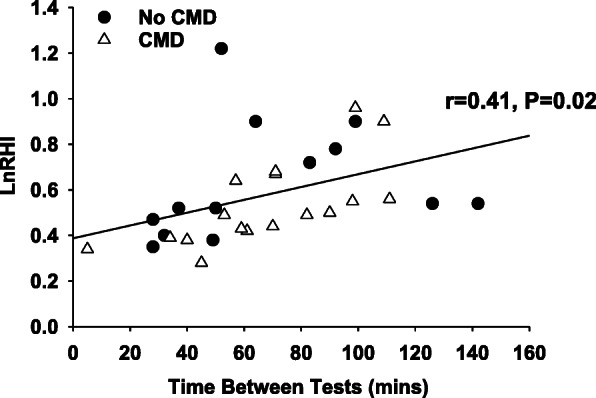
Correlation between the natural logarithm of the reactive hyperemia index (LnRHI) and the time between the graded exercise stress test and the vascular assessment in patients who exercised prior to the vascular assessment. Black circles represent patients without coronary microvascular dysfunction (CMD). White triangles represent patients with CMD

Prior to the adjustment for pharmaceutical use and fasting, the RHI and LnRHI were lower in patients with confirmed CMD compared to patients without CMD (*P* < 0.01), and exercisers demonstrated an attenuated RHI and LnRHI compared to non-exercisers (*P* < 0.01; Table 4). Augmentation indices (AI and AI@75 bpm) were not different between patients with and without CMD (*P* > 0.05), and exercisers demonstrated an attenuated AI compared to non-exercisers (*P* < 0.01), while the AI@75 bpm were not different between exercisers and non-exercisers (*P* > 0.05, Table 4). After adjusting for pharmaceutical use and fasting, the RHI and LnRHI were still lower in patients with confirmed CMD compared to patients without CMD (*P* < 0.01; Fig. 2). Further, RHI, LnRHI, and AI were still lower in exercisers compared to non-exercisers (P < 0.01; Fig. 2), though there were no exercise or condition effects in the adjusted AI@75 bpm (*P* > 0.05; Fig. 3). Correlations of the time between tests and the LnRHI were conducted for all exercisers combined, and a positive correlation was observed (r = 0.54, *P* < 0.01; Fig. 4). Further, in patients who did not exercise prior to the vascular assessment, the ROC_AUC_ for LnRHI to predict CMD was 0.76 (95% CI: 0.62–0.91; *P* < 0.01). However, in patients who performed exercise prior to the vascular assessment, the ROC_AUC_ for LnRHI to predict CMD was 0.60 (95% CI: 0.40–0.81; *P* = 0.34). Lastly, covariate analysis of the adjusted model (data not shown) found that there were no effects of anti-hypertensive medication use, anti-cholesterol medication use, or fasted state on RHI, LnRHI, AI, or AI@75 bpm (*P* > 0.05).

**Table 4:** Unadjusted reactive hyperemia index (RHI), natural logarithm of the RHI (LnRHI), augmentation index (AI), and augmentation index at 75 bpm (AI@75 bpm) in patients with or without coronary microvascular dysfunction (CMD). Patients were separated into groups who did and did not exercise prior to vascular assessment. Mean ± SD

	No CMD		CMD	
	Non-EX	Exercisers	Non-EX	Exercisers
RHI^b^	2.41 ± 0.55	1.95 ± 0.56^a^	1.93 ± 0.53	1.74 ± 0.35^a^
LnRHI^b^	0.85 ± 0.23	0.63 ± 0.26^a^	0.63 ± 0.25	0.54 ± 0.19^a^
AI	25.2 ± 19.1	8.5 ± 17.8^a^	23.7 ± 25.3	13.7 ± 16.4^a^
AI@75 bpm	16.9 ± 16.6	8.1 ± 13.6	17.4 ± 25.8	12.0 ± 14.0

*Non-EX* Non-Exercisers, *CMD* Coronary Microvascular Dysfunction, *RHI* Reactive Hyperemia Index, *LnRHI* Natural logarithm of the RHI, *AI* Augmentation Index, *AI@75 bpm* Augmentation Index at 75 bpm. ^a^ indicates a significant exercise effect. ^b^ indicates a significant effect of CMD. Mean ± SD

## Discussion

The effect of acute exercise on peripheral microvascular function in patients with suspected coronary microvascular dysfunction (CMD) was previously unknown. First, in support of our recent findings [6], patients with CMD demonstrated impaired peripheral microvascular function. Second, consistent with studies in conduit arteries and healthy participants [14], acute exercise impaired microvascular function in patients with and without CMD. Third, contrary to our hypothesis, acute maximal exercise impaired peripheral microvascular function equally in patients with and without CMD. Fourth, acute graded exercise stress testing abolishes the capacity for RH-PAT to predict the presence of CMD in patients with chest pain and non-obstructive coronary arteries.

Most studies investigating vascular function following acute exercise have utilized brachial artery flow-mediated dilation as a measure of conduit artery endothelial function. Using this method, post-exercise endothelial function has been reported inconsistently, as both increases [26–28] and decreases [27, 29–31] in brachial artery endothelial function have been observed. Inconsistent findings between studies could be due to differing intensity of exercise. Specifically, in young healthy participants, studies reporting decreased brachial artery endothelial function following exercise commonly utilize higher intensity exercise [31], while improved brachial artery endothelial function is commonly observed during low to moderate intensity exercise [26]. Additionally, the health/fitness status of participants may also influence the post-exercise vascular response. For instance, resistance exercise attenuated microvascular function in sedentary participants, though this was not observed in trained participants [32]. Similar findings in overweight participants have been observed, such that active participants increased, and inactive participants decreased, brachial artery endothelial function following acute exercise [27]. Patients with intermittent claudication also reported attenuated brachial artery endothelial function following higher intensity treadmill exercise [29]. In the current study, suspected CMD patients who conducted symptom-limited maximal treadmill exercise prior to RH-PAT testing exhibited an attenuation of RHI and LnRHI, supporting previous work that both higher intensity exercise, and patients with cardiovascular disease demonstrate attenuated peripheral endothelial function.

The mechanisms responsible for reducing microvascular function in the current study are unclear. Previous investigators have proposed that attenuated endothelial function due to the performance of exercise may be a result of elevated resting conduit artery diameter and blood flow [14]. This observation is particularly relevant considering that brachial artery diameter has been shown to influence the RH-PAT response, such that a larger brachial artery diameter is associated with a higher baseline pulse amplitude and a lower RHI [33]. Therefore, these post-exercise vascular alterations may act to diminish the vasodilatory reservoir and mathematically reduce the RH-PAT response. Alternatively, oxidative stress is increased during high intensity exercise [34, 35], facilitating a reduction in endothelial function following acute exercise in health [27, 36] and disease [29]. Since oxidative stress has been shown to be elevated in patients with CMD [37], we had hypothesized that baseline impairments in peripheral endothelial function in CMD patients would exacerbate post-exercise microvascular function compared to the non-CMD group, yet this was not observed. We suggest that measurements of oxidative stress should be added to future longitudinal investigations of the effect of exercise in CMD. Lastly, RH-PAT is strongly associated with resting sympathetic activity [38], to a greater extent than brachial artery flow mediated dilation [39]. Given that sympathetic activity is elevated following exercise, the reduced RHI and LnRHI in the current study may potentially be related to increased sympathetic activity, rather than reduced endothelial function caused by oxidative stress.

Contrary to previous reports [40, 41], the present study observed that measures of arterial stiffness were not different in patients with and without CMD. Several studies observed that both the aortic augmentation index [40] and the carotid artery augmentation index [41] were associated with the CFR response to intravenous adenosine in patients with CMD, implying that arterial stiffness in the large, mainly elastic arteries, is elevated in patients with CMD. In the current study, augmentation index was obtained from the finger microvasculature using the EndoPAT device, suggesting that arterial stiffness in the peripheral vasculature is similar in both conditions. We did not observe a significant effect of exercise on augmentation index normalized to heart rate (AI@75 bpm) indicating that acute exercise did not influence peripheral arterial stiffness in patients with suspected CMD. The validity of the augmentation indices obtained from the EndoPAT device have yet to be compared to a gold-standard measurement such as applanation tonometry of conduit vessels.

### Clinical relevance

The use of RH-PAT is a clinically appealing method of measuring peripheral microvascular function, given that RH-PAT assessments are relatively cost efficient, operator independent, possess objective and automated analyses [2], and are highly reproducible [42, 43]. However, despite growing interest, the standardizations for RH-PAT assessments have not been determined in health or disease relative to other established measures of vascular function, such as flow-mediated dilation [12, 13]. Further, flow-mediated dilation and RH-PAT are poorly correlated [15] and can be influenced differently by aging [15], exercise training [44], and medication use [16], suggesting that established variables known to alter flow-mediated dilation do not necessarily alter RH-PAT. These observations further exemplify the importance of establishing RH-PAT specific standardizations. Our data suggest that RHI and LnRHI are reduced by standard exercise stress testing, abolishing the predictive capacity of RH-PAT in identifying patients with CMD. Notably, the deterioration of RH-PAT due to CMD and the deterioration due to exercise in patients without CMD were similar. Therefore, caution should be used in interpreting RH-PAT results when completed after exercise stress testing.

Additionally, our data show that RHI and LnRHI were not influenced by pharmacological use or fasting. In particular, the observation that fasting did not demonstrate a significant influence is important for CMD patients since fasting prior to exercise could lead to hypoglycemia during their clinical visit and/or exercise stress test, given the high prevalence of diabetes mellitus [45, 46].

### Limitations

We acknowledge several important considerations. First, this study was a cross-sectional study design. To fully elucidate the relationship between RHI and acute exercise, a repeated measures study design should be completed. To date, no study has investigated the relationship between RHI and exercise in patients with suspected CMD, and these results provide strong evidence to continue this work. Second, since the time delay between the RH-PAT assessment and the diagnostic coronary reactivity testing was ~ 5 months, we cannot rule out that peripheral vascular function could have been altered within this time period due to changes in lifestyle or severity of condition, despite medication use being unchanged. However, the relationship between peripheral vascular function and the presence of CMD would likely have been lessened due to the time delay, decreasing the likelihood of finding a significant difference. Thirdly, although patients with and without CMD had similar exercise tolerances during the GXT (suggesting equal levels of cardiorespiratory fitness), this study did not objectively assess physical activity or oxygen consumption via a full cardiopulmonary exercise test. Lastly, lower physical activity has been linked with impaired endothelial function following acute exercise [30] potentially influencing our results. Therefore, future studies should obtain accurate measurements of fitness using accelerometry and oxygen consumption.

## Conclusions

These data support previous findings that patients with confirmed CMD have impaired peripheral microvascular function compared to patients without CMD and are the first data to suggest that exercise stress testing impairs peripheral microvascular function in all patients with suspected CMD. Further, in exercisers with and without CMD, there was a positive relationship between peripheral microvascular function and the time between exercise testing and vascular assessment. We recommend that RH-PAT measurements should not be conducted after exercise has been performed.

## Data Availability

The datasets used and analyzed during the current study are available from the principal researcher (HE: edgell@yorku.ca) upon reasonable request.

## Abbreviations

AI: Augmentation index
AI@75 bpm: Augmentation index at 75 bpm
CFR: Coronary flow reserve,
CMD: Coronary microvascular dysfunction
DBP: Diastolic blood pressure
FFR: Fractional flow reserve
GXT: Graded exercise stress test
HR: Heart rate
IMR: Index of microvascular resistance
LBBB: Left bundle branch block
LnRHI: Natural logarithm of the reactive hyperemia index
Pa: Pressure at proximal coronary segment
Pd: Pressure at distal coronary segment
RH-PAT: Reactive hyperemia peripheral arterial tonometry
RHI: Reactive hyperemia index
SBP: Systolic blood pressure
Tmn: Mean transit time
